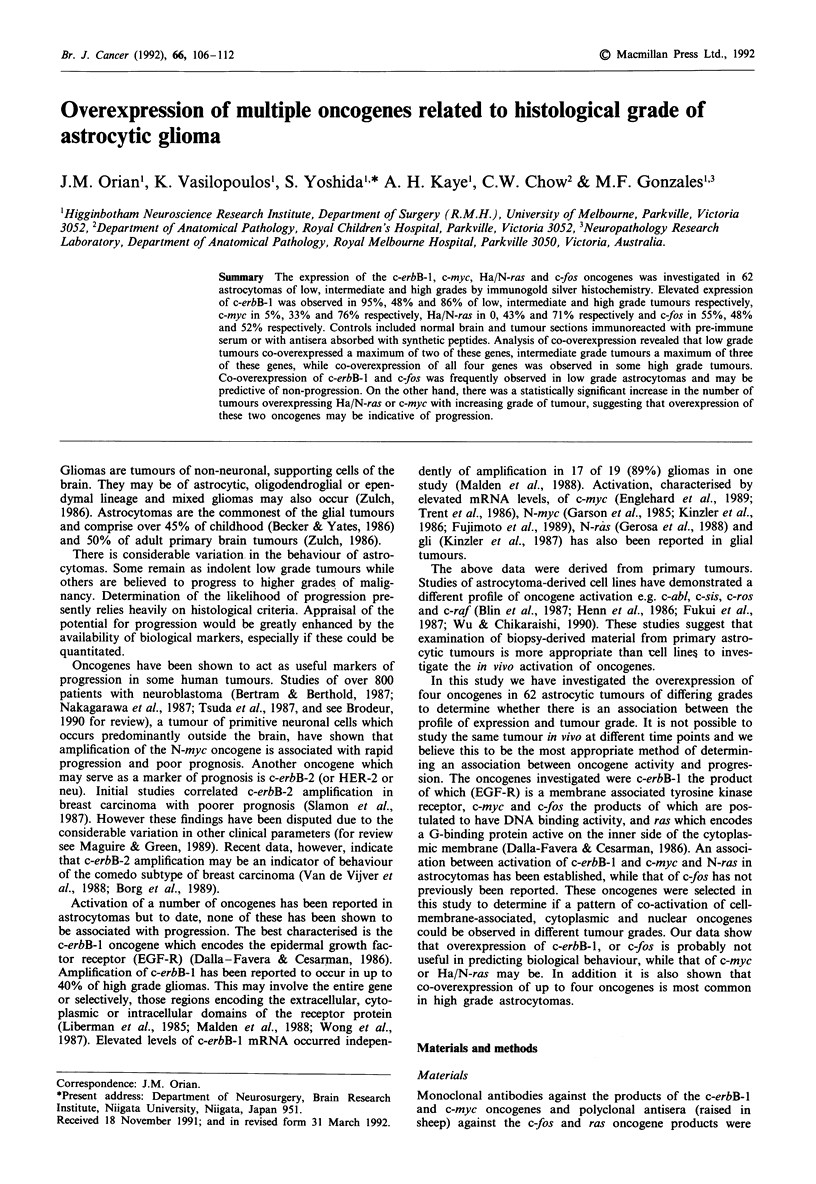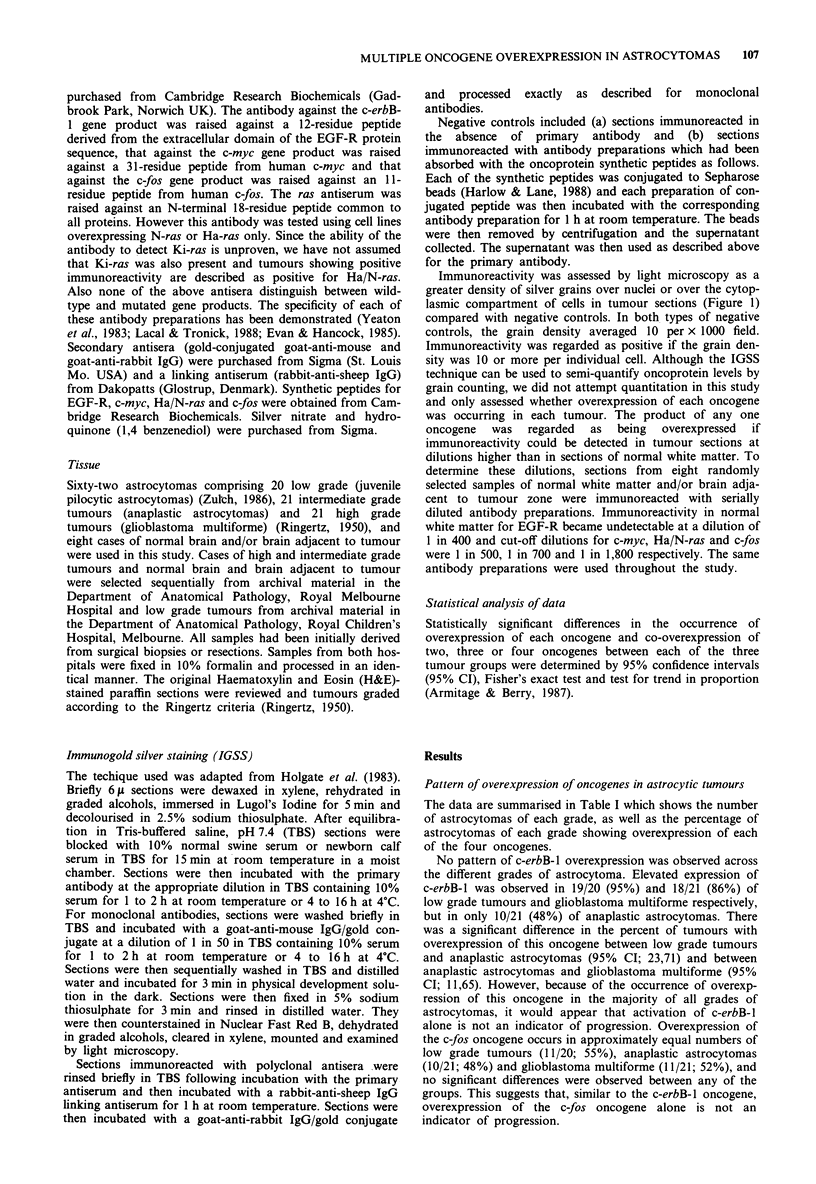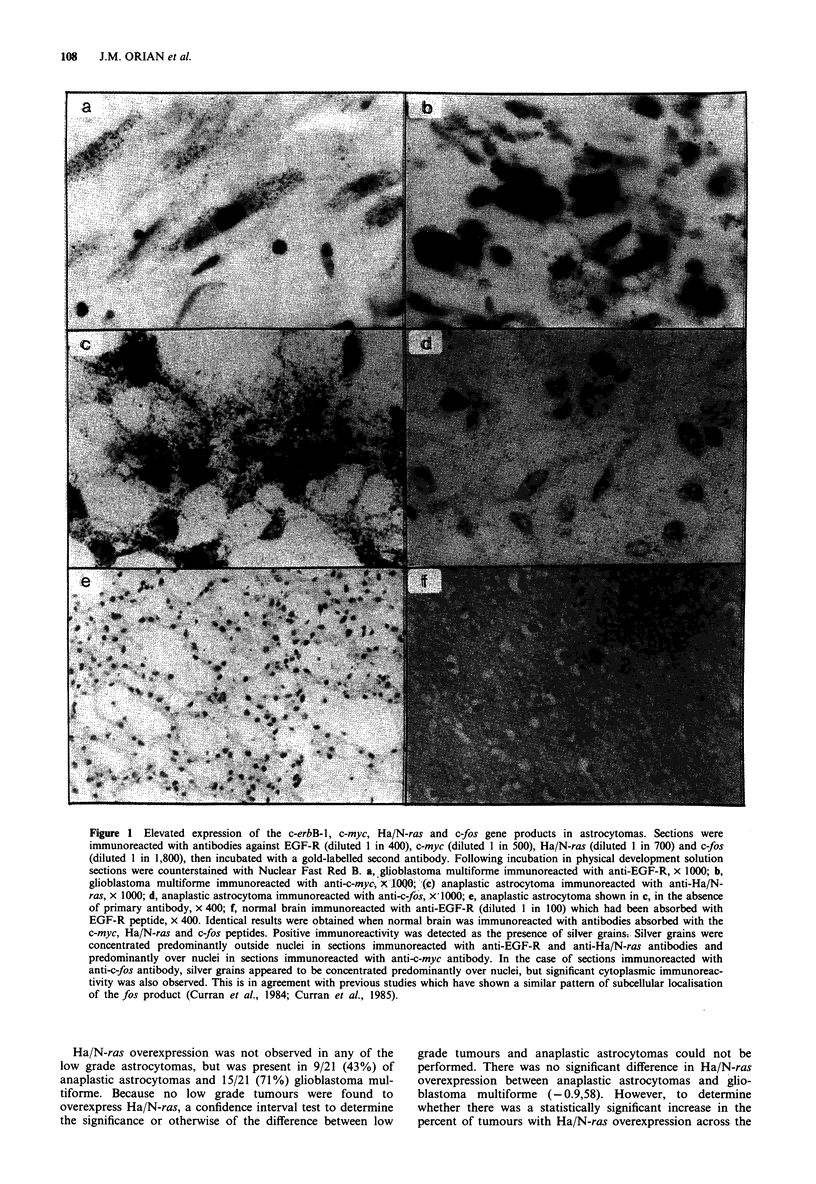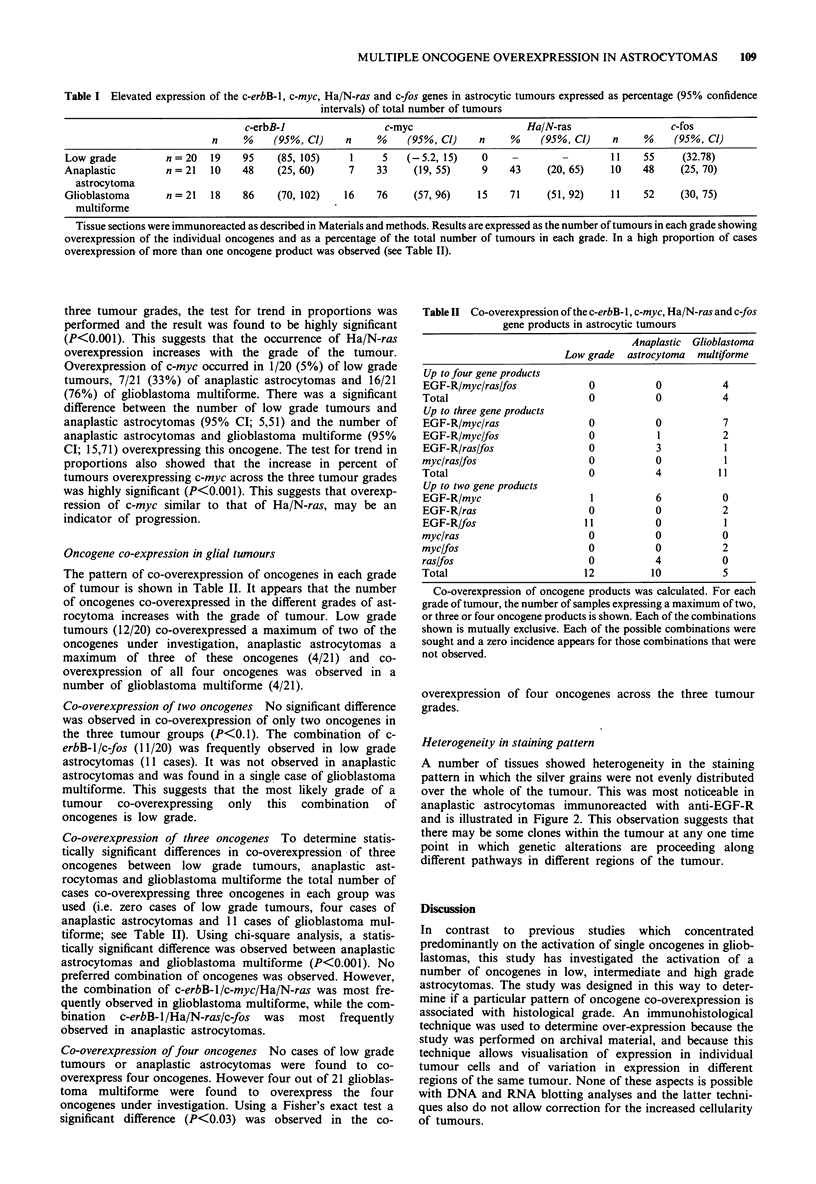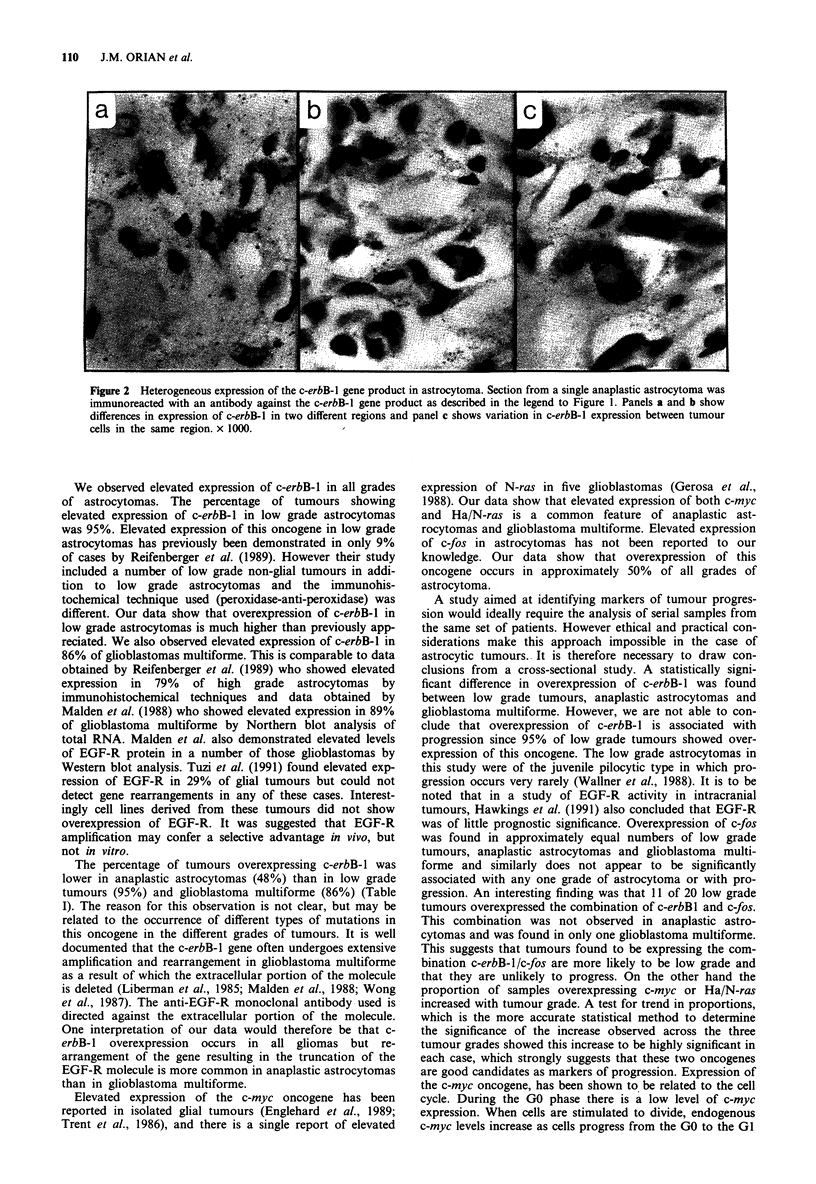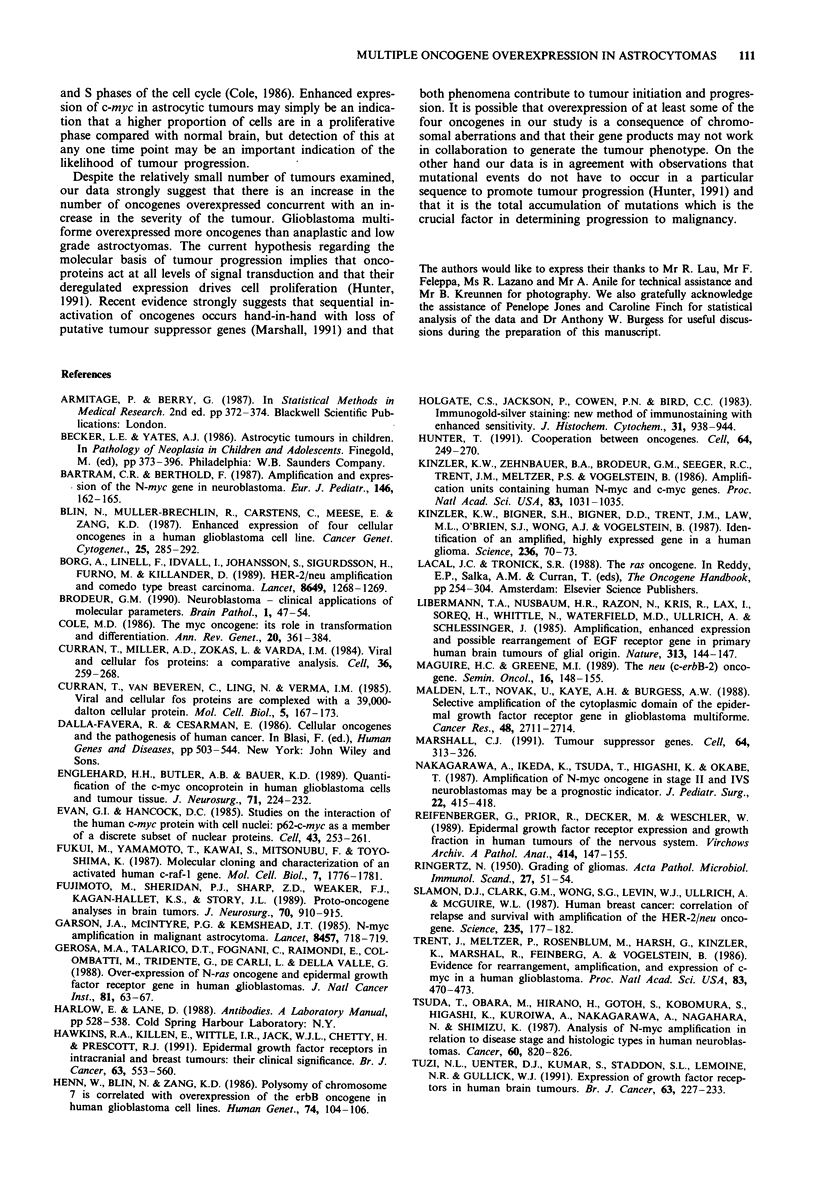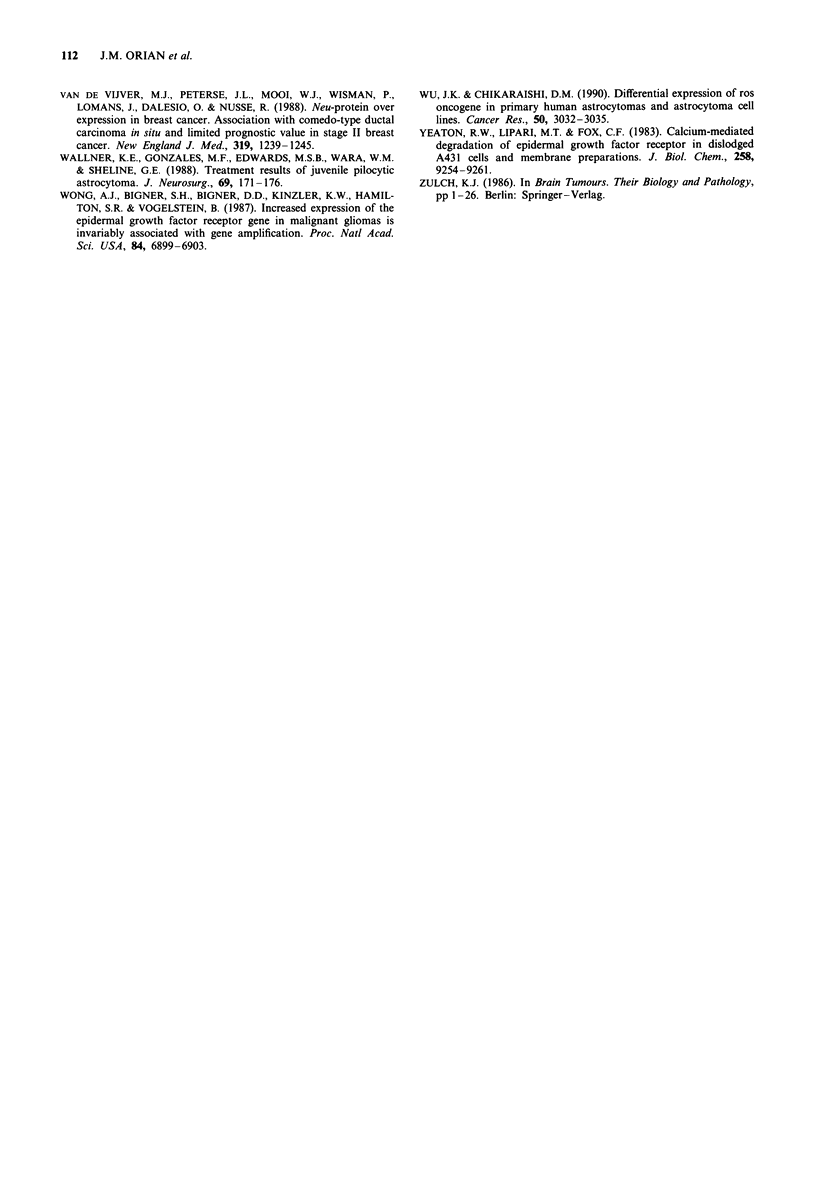# Overexpression of multiple oncogenes related to histological grade of astrocytic glioma.

**DOI:** 10.1038/bjc.1992.225

**Published:** 1992-07

**Authors:** J. M. Orian, K. Vasilopoulos, S. Yoshida, A. H. Kaye, C. W. Chow, M. F. Gonzales

**Affiliations:** Higginbotham Neuroscience Research Institute, Department of Surgery (R.M.H.), University of Melbourne, Parkville, Victoria.

## Abstract

**Images:**


Br. J. Cancer (1992), 66, 106-112 © Macmillan Press Ltd., 1992

### Overexpression of multiple oncogenes related to histological grade of astrocytic glioma

J.M. Orian[1], K. Vasilopoulos[1], S. Yoshida[1,]* A. H. Kaye[1], C.W. Chow[2] & M.F. Gonzales[1,3]

[1]*Higginbotham Neuroscience Research Institute, Department of Surgery (R.M.H.), University of Melbourne, Parkville, Victoria 3052,* [2]*Department of Anatomical Pathology, Royal Children's Hospital, Parkville, Victoria 3052,* [3]*Neuropathology Research Laboratory, Department of Anatomical Pathology, Royal Melbourne Hospital, Parkville 3050, Victoria, Australia.*

**Summary** The expression of the c-*erb*B-1, c-*myc*, Ha/N-*ras* and c-*fos* oncogenes was investigated in 62 astrocytomas of low, intermediate and high grades by immunogold silver histochemistry. Elevated expression of c-*erb*B-1 was observed in 95%, 48% and 86% of low, intermediate and high grade tumours respectively, c-*myc* in 5%, 33% and 76% respectively, Ha/N-*ras* in 0, 43% and 71% respectively and c-*fos* in 55%, 48% and 52% respectively. Controls included normal brain and tumour sections immunoreacted with pre-immune serum or with antisera absorbed with synthetic peptides. Analysis of co-overexpression revealed that low grade tumours co-overexpressed a maximum of two of these genes, intermediate grade tumours a maximum of three of these genes, while co-overexpression of all four genes was observed in some high grade tumours. Co-overexpression of c-*erb*B-1 and c-*fos* was frequently observed in low grade astrocytomas and may be predictive of non-progression. On the other hand, there was a statistically significant increase in the number of tumours overexpressing Ha/N-*ras* or c-*myc* with increasing grade of tumour, suggesting that overexpression of these two oncogenes may be indicative of progression.

Gliomas are tumours of non-neuronal, supporting cells of the brain. They may be of astrocytic, oligodendroglial or ependymal lineage and mixed gliomas may also occur (Zulch, 1986). Astrocytomas are the commonest of the glial tumours and comprise over 45% of childhood (Becker & Yates, 1986) and 50% of adult primary brain tumours (Zulch, 1986).

There is considerable variation in the behaviour of astrocytomas. Some remain as indolent low grade tumours while others are believed to progress to higher grades of malignancy. Determination of the likelihood of progression presently relies heavily on histological criteria. Appraisal of the potential for progression would be greatly enhanced by the availability of biological markers, especially if these could be quantitated.

Oncogenes have been shown to act as useful markers of progression in some human tumours. Studies of over 800 patients with neuroblastoma (Bertram & Berthold, 1987; Nakagarawa *et al.*, 1987; Tsuda *et al.*, 1987, and see Brodeur, 1990 for review), a tumour of primitive neuronal cells which occurs predominantly outside the brain, have shown that amplification of the N-*myc* oncogene is associated with rapid progression and poor prognosis. Another oncogene which may serve as a marker of prognosis is c-*erb*B-2 (or HER-2 or neu). Initial studies correlated c-*erb*B-2 amplification in breast carcinoma with poorer prognosis (Slamon *et al.*, 1987). However these findings have been disputed due to the considerable variation in other clinical parameters (for review see Maguire & Green, 1989). Recent data, however, indicate that c-*erb*B-2 amplification may be an indicator of behaviour of the comedo subtype of breast carcinoma (Van de Vijver *et al.*, 1988; Borg *et al.*, 1989).

Activation of a number of oncogenes has been reported in astrocytomas but to date, none of these has been shown to be associated with progression. The best characterised is the c-*erb*B-1 oncogene which encodes the epidermal growth factor receptor (EGF-R) (Dalla-Favera & Cesarman, 1986). Amplification of c-*erb*B-1 has been reported to occur in up to 40% of high grade gliomas. This may involve the entire gene or selectively, those regions encoding the extracellular, cytoplasmic or intracellular domains of the receptor protein (Liberman *et al.*, 1985; Malden *et al.*, 1988; Wong *et al.*, 1987). Elevated levels of c-*erb*B-1 mRNA occurred independently of amplification in 17 of 19 (89%) gliomas in one study (Malden *et al.*, 1988). Activation, characterised by elevated mRNA levels, of c-*myc* (Englehard *et al.*, 1989; Trent *et al.*, 1986), N-*myc* (Garson *et al.*, 1985; Kinzler *et al.*, 1986; Fujimoto *et al.*, 1989), N-*ras* (Gerosa *et al.*, 1988) and gli (Kinzler *et al.*, 1987) has also been reported in glial tumours.

The above data were derived from primary tumours. Studies of astrocytoma-derived cell lines have demonstrated a different profile of oncogene activation e.g. c-*abl*, c-*sis*, c-*ros* and c-*raf* (Blin *et al.*, 1987; Henn *et al.*, 1986; Fukui *et al.*, 1987; Wu & Chikaraishi, 1990). These studies suggest that examination of biopsy-derived material from primary astrocytic tumours is more appropriate than cell lines to investigate the *in vivo* activation of oncogenes.

In this study we have investigated the overexpression of four oncogenes in 62 astrocytic tumours of differing grades to determine whether there is an association between the profile of expression and tumour grade. It is not possible to study the same tumour *in vivo* at different time points and we believe this to be the most appropriate method of determining an association between oncogene activity and progression. The oncogenes investigated were c-*erb*B-1 the product of which (EGF-R) is a membrane associated tyrosine kinase receptor, c-*myc* and c-*fos* the products of which are postulated to have DNA binding activity, and *ras* which encodes a G-binding protein active on the inner side of the cytoplasmic membrane (Dalla-Favera & Cesarman, 1986). An association between activation of c-*erb*B-1 and c-*myc* and N-*ras* in astrocytomas has been established, while that of c-*fos* has not previously been reported. These oncogenes were selected in this study to determine if a pattern of co-activation of cell-membrane-associated, cytoplasmic and nuclear oncogenes could be observed in different tumour grades. Our data show that overexpression of c-*erb*B-1, or c-*fos* is probably not useful in predicting biological behaviour, while that of c-*myc* or Ha/N-*ras* may be. In addition it is also shown that co-overexpression of up to four oncogenes is most common in high grade astrocytomas.

### Materials and methods

### *Materials*

Monoclonal antibodies against the products of the c-*erb*B-1 and c-*myc* oncogenes and polyclonal antisera (raised in sheep) against the c-*fos* and *ras* oncogene products were

Correspondence: J.M. Orian.
*Present address: Department of Neurosurgery, Brain Research Institute, Niigata University, Niigata, Japan 951.
Received 18 November 1991; and in revised form 31 March 1992.

purchased from Cambridge Research Biochemicals (Gadbrook Park, Norwich UK). The antibody against the c-*erb*B-1 gene product was raised against a 12-residue peptide derived from the extracellular domain of the EGF-R protein sequence, that against the c-*myc* gene product was raised against a 31-residue peptide from human c-*myc* and that against the c-*fos* gene product was raised against an 11-residue peptide from human c-*fos*. The *ras* antiserum was raised against an N-terminal 18-residue peptide common to all proteins. However this antibody was tested using cell lines overexpressing N-*ras* or Ha-*ras* only. Since the ability of the antibody to detect Ki-*ras* is unproven, we have not assumed that Ki-*ras* was also present and tumours showing positive immunoreactivity are described as positive for Ha/N-*ras*. Also none of the above antisera distinguish between wild-type and mutated gene products. The specificity of each of these antibody preparations has been demonstrated (Yeaton *et al.*, 1983; Lacal & Tronick, 1988; Evan & Hancock, 1985). Secondary antisera (gold-conjugated goat-anti-mouse and goat-anti-rabbit IgG) were purchased from Sigma (St. Louis Mo. USA) and a linking antiserum (rabbit-anti-sheep IgG) from Dakopatts (Glostrup, Denmark). Synthetic peptides for EGF-R, c-*myc*, Ha/N-*ras* and c-*fos* were obtained from Cambridge Research Biochemicals. Silver nitrate and hydroquinone (1,4 benzenediol) were purchased from Sigma.

### *Tissue*

Sixty-two astrocytomas comprising 20 low grade (juvenile pilocytic astrocytomas) (Zulch, 1986), 21 intermediate grade tumours (anaplastic astrocytomas) and 21 high grade tumours (glioblastoma multiforme) (Ringertz, 1950), and eight cases of normal brain and/or brain adjacent to tumour were used in this study. Cases of high and intermediate grade tumours and normal brain and brain adjacent to tumour were selected sequentially from archival material in the Department of Anatomical Pathology, Royal Melbourne Hospital and low grade tumours from archival material in the Department of Anatomical Pathology, Royal Children's Hospital, Melbourne. All samples had been initially derived from surgical biopsies or resections. Samples from both hospitals were fixed in 10% formalin and processed in an identical manner. The original Haematoxylin and Eosin (H&E)-stained paraffin sections were reviewed and tumours graded according to the Ringertz criteria (Ringertz, 1950).

### *Immunogold silver staining (IGSS)*

The techique used was adapted from Holgate *et al.* (1983). Briefly 6 μ sections were dewaxed in xylene, rehydrated in graded alcohols, immersed in Lugol's Iodine for 5 min and decolourised in 2.5% sodium thiosulphate. After equilibration in Tris-buffered saline, pH 7.4 (TBS) sections were blocked with 10% normal swine serum or newborn calf serum in TBS for 15 min at room temperature in a moist chamber. Sections were then incubated with the primary antibody at the appropriate dilution in TBS containing 10% serum for 1 to 2 h at room temperature or 4 to 16 h at 4°C. For monoclonal antibodies, sections were washed briefly in TBS and incubated with a goat-anti-mouse IgG/gold conjugate at a dilution of 1 in 50 in TBS containing 10% serum for 1 to 2 h at room temperature or 4 to 16 h at 4°C. Sections were then sequentially washed in TBS and distilled water and incubated for 3 min in physical development solution in the dark. Sections were then fixed in 5% sodium thiosulphate for 3 min and rinsed in distilled water. They were then counterstained in Nuclear Fast Red B, dehydrated in graded alcohols, cleared in xylene, mounted and examined by light microscopy.

Sections immunoreacted with polyclonal antisera were rinsed briefly in TBS following incubation with the primary antiserum and then incubated with a rabbit-anti-sheep IgG linking antiserum for 1 h at room temperature. Sections were then incubated with a goat-anti-rabbit IgG/gold conjugate and processed exactly as described for monoclonal antibodies.

Negative controls included (a) sections immunoreacted in the absence of primary antibody and (b) sections immunoreacted with antibody preparations which had been absorbed with the oncoprotein synthetic peptides as follows. Each of the synthetic peptides was conjugated to Sepharose beads (Harlow & Lane, 1988) and each preparation of conjugated peptide was then incubated with the corresponding antibody preparation for 1 h at room temperature. The beads were then removed by centrifugation and the supernatant collected. The supernatant was then used as described above for the primary antibody.

Immunoreactivity was assessed by light microscopy as a greater density of silver grains over nuclei or over the cytoplasmic compartment of cells in tumour sections (Figure 1) compared with negative controls. In both types of negative controls, the grain density averaged 10 per × 1000 field. Immunoreactivity was regarded as positive if the grain density was 10 or more per individual cell. Although the IGSS technique can be used to semi-quantify oncoprotein levels by grain counting, we did not attempt quantitation in this study and only assessed whether overexpression of each oncogene was occurring in each tumour. The product of any one oncogene was regarded as being overexpressed if immunoreactivity could be detected in tumour sections at dilutions higher than in sections of normal white matter. To determine these dilutions, sections from eight randomly selected samples of normal white matter and/or brain adjacent to tumour zone were immunoreacted with serially diluted antibody preparations. Immunoreactivity in normal white matter for EGF-R became undetectable at a dilution of 1 in 400 and cut-off dilutions for c-*myc*, Ha/N-*ras* and c-*fos* were 1 in 500, 1 in 700 and 1 in 1,800 respectively. The same antibody preparations were used throughout the study.

### *Statistical analysis of data*

Statistically significant differences in the occurrence of overexpression of each oncogene and co-overexpression of two, three or four oncogenes between each of the three tumour groups were determined by 95% confidence intervals (95% CI), Fisher's exact test and test for trend in proportion (Armitage & Berry, 1987).

### Results

### *Pattern of overexpression of oncogenes in astrocytic tumours*

The data are summarised in Table I which shows the number of astrocytomas of each grade, as well as the percentage of astrocytomas of each grade showing overexpression of each of the four oncogenes.

No pattern of c-*erb*B-1 overexpression was observed across the different grades of astrocytoma. Elevated expression of c-*erb*B-1 was observed in 19/20 (95%) and 18/21 (86%) of low grade tumours and glioblastoma multiforme respectively, but in only 10/21 (48%) of anaplastic astrocytomas. There was a significant difference in the percent of tumours with overexpression of this oncogene between low grade tumours and anaplastic astrocytomas (95% CI; 23,71) and between anaplastic astrocytomas and glioblastoma multiforme (95% CI; 11,65). However, because of the occurrence of overexpression of this oncogene in the majority of all grades of astrocytomas, it would appear that activation of c-*erb*B-1 alone is not an indicator of progression. Overexpression of the c-*fos* oncogene occurs in approximately equal numbers of low grade tumours (11/20; 55%), anaplastic astrocytomas (10/21; 48%) and glioblastoma multiforme (11/21; 52%), and no significant differences were observed between any of the groups. This suggests that, similar to the c-*erb*B-1 oncogene, overexpression of the c-*fos* oncogene alone is not an indicator of progression.

**Figure 1** Elevated expression of the c-*erb*B-1, c-*myc*, Ha/N-*ras* and c-*fos* gene products in astrocytomas. Sections were immunoreacted with antibodies against EGF-R (diluted 1 in 400), c-*myc* (diluted 1 in 500), Ha/N-*ras* (diluted 1 in 700) and c-*fos* (diluted 1 in 1,800), then incubated with a gold-labelled second antibody. Following incubation in physical development solution sections were counterstained with Nuclear Fast Red B. **a**, glioblastoma multiforme immunoreacted with anti-EGF-R, × 1000; **b**, glioblastoma multiforme immunoreacted with anti-c-*myc*, × 1000; (**c**) anaplastic astrocytoma immunoreacted with anti-Ha/N-*ras*, × 1000; **d**, anaplastic astrocytoma immunoreacted with anti-c-*fos*, × 1000; **e**, anaplastic astrocytoma shown in **c**, in the absence of primary antibody, × 400; **f**, normal brain immunoreacted with anti-EGF-R (diluted 1 in 100) which had been absorbed with EGF-R peptide, × 400. Identical results were obtained when normal brain was immunoreacted with antibodies absorbed with the c-*myc*, Ha/N-*ras* and c-*fos* peptides. Positive immunoreactivity was detected as the presence of silver grains. Silver grains were concentrated predominantly outside nuclei in sections immunoreacted with anti-EGF-R and anti-Ha/N-*ras* antibodies and predominantly over nuclei in sections immunoreacted with anti-c-*myc* antibody. In the case of sections immunoreacted with anti-c-*fos* antibody, silver grains appeared to be concentrated predominantly over nuclei, but significant cytoplasmic immunoreactivity was also observed. This is in agreement with previous studies which have shown a similar pattern of subcellular localisation of the *fos* product (Curran *et al.*, 1984; Curran *et al.*, 1985).

Ha/N-*ras* overexpression was not observed in any of the low grade astrocytomas, but was present in 9/21 (43%) of anaplastic astrocytomas and 15/21 (71%) glioblastoma multiforme. Because no low grade tumours were found to overexpress Ha/N-*ras*, a confidence interval test to determine the significance or otherwise of the difference between low grade tumours and anaplastic astrocytomas could not be performed. There was no significant difference in Ha/N-*ras* overexpression between anaplastic astrocytomas and glioblastoma multiforme (−0.9,58). However, to determine whether there was a statistically significant increase in the percent of tumours with Ha/N-*ras* overexpression across the

**Table I** Elevated expression of the c-*erb*B-1, c-*myc*, Ha/N-*ras* and c-*fos* genes in astrocytic tumours expressed as percentage (95% confidence intervals) of total number of tumours

| | | *c*-erb*B-1* | | | *c*-myc | | | *Ha/N*-ras | | | c-fos | | |
|---|---|---|---|---|---|---|---|---|---|---|---|---|---|
| | | *n* | *%* | *(95%, CI)* | *n* | *%* | *(95%, CI)* | *n* | *%* | *(95%, CI)* | *n* | *%* | *(95%, CI)* |
| Low grade | *n* = 20 | 19 | 95 | (85, 105) | 1 | 5 | (−5.2, 15) | 0 | – | – | 11 | 55 | (32.78) |
| Anaplastic astrocytoma | *n* = 21 | 10 | 48 | (25, 60) | 7 | 33 | (19, 55) | 9 | 43 | (20, 65) | 10 | 48 | (25, 70) |
| Glioblastoma multiforme | *n* = 21 | 18 | 86 | (70, 102) | 16 | 76 | (57, 96) | 15 | 71 | (51, 92) | 11 | 52 | (30, 75) |

Tissue sections were immunoreacted as described in Materials and methods. Results are expressed as the number of tumours in each grade showing overexpression of the individual oncogenes and as a percentage of the total number of tumours in each grade. In a high proportion of cases overexpression of more than one oncogene product was observed (see Table II).

three tumour grades, the test for trend in proportions was performed and the result was found to be highly significant ($P<0.001$). This suggests that the occurrence of Ha/N-*ras* overexpression increases with the grade of the tumour. Overexpression of c-*myc* occurred in 1/20 (5%) of low grade tumours, 7/21 (33%) of anaplastic astrocytomas and 16/21 (76%) of glioblastoma multiforme. There was a significant difference between the number of low grade tumours and anaplastic astrocytomas (95% CI; 5,51) and the number of anaplastic astrocytomas and glioblastoma multiforme (95% CI; 15,71) overexpressing this oncogene. The test for trend in proportions also showed that the increase in percent of tumours overexpressing c-*myc* across the three tumour grades was highly significant ($P<0.001$). This suggests that overexpression of c-*myc* similar to that of Ha/N-*ras*, may be an indicator of progression.

### *Oncogene co-expression in glial tumours*

The pattern of co-overexpression of oncogenes in each grade of tumour is shown in Table II. It appears that the number of oncogenes co-overexpressed in the different grades of astrocytoma increases with the grade of tumour. Low grade tumours (12/20) co-overexpressed a maximum of two of the oncogenes under investigation, anaplastic astrocytomas a maximum of three of these oncogenes (4/21) and co-overexpression of all four oncogenes was observed in a number of glioblastoma multiforme (4/21).

*Co-overexpression of two oncogenes* No significant difference was observed in co-overexpression of only two oncogenes in the three tumour groups ($P<0.1$). The combination of c-*erb*B-1/c-*fos* (11/20) was frequently observed in low grade astrocytomas (11 cases). It was not observed in anaplastic astrocytomas and was found in a single case of glioblastoma multiforme. This suggests that the most likely grade of a tumour co-overexpressing only this combination of oncogenes is low grade.

*Co-overexpression of three oncogenes* To determine statistically significant differences in co-overexpression of three oncogenes between low grade tumours, anaplastic astrocytomas and glioblastoma multiforme the total number of cases co-overexpressing three oncogenes in each group was used (i.e. zero cases of low grade tumours, four cases of anaplastic astrocytomas and 11 cases of glioblastoma multiforme; see Table II). Using chi-square analysis, a statistically significant difference was observed between anaplastic astrocytomas and glioblastoma multiforme ($P<0.001$). No preferred combination of oncogenes was observed. However, the combination of c-*erb*B-1/c-*myc*/Ha/N-*ras* was most frequently observed in glioblastoma multiforme, while the combination c-*erb*B-1/Ha/N-*ras*/c-*fos* was most frequently observed in anaplastic astrocytomas.

*Co-overexpression of four oncogenes* No cases of low grade tumours or anaplastic astrocytomas were found to co-overexpress four oncogenes. However four out of 21 glioblastoma multiforme were found to overexpress the four oncogenes under investigation. Using a Fisher's exact test a significant difference ($P<0.03$) was observed in the co-overexpression of four oncogenes across the three tumour grades.

**Table II** Co-overexpression of the c-*erb*B-1, c-*myc*, Ha/N-*ras* and c-*fos* gene products in astrocytic tumours

| | *Low grade* | *Anaplastic astrocytoma* | *Glioblastoma multiforme* |
|---|---|---|---|
| *Up to four gene products* | | | |
| EGF-R/*myc*/*ras*/*fos* | 0 | 0 | 4 |
| Total | 0 | 0 | 4 |
| *Up to three gene products* | | | |
| EGF-R/*myc*/*ras* | 0 | 0 | 7 |
| EGF-R/*myc*/*fos* | 0 | 1 | 2 |
| EGF-R/*ras*/*fos* | 0 | 3 | 1 |
| *myc*/*ras*/*fos* | 0 | 0 | 1 |
| Total | 0 | 4 | 11 |
| *Up to two gene products* | | | |
| EGF-R/*myc* | 1 | 6 | 0 |
| EGF-R/*ras* | 0 | 0 | 2 |
| EGF-R/*fos* | 11 | 0 | 1 |
| *myc*/*ras* | 0 | 0 | 0 |
| *myc*/*fos* | 0 | 0 | 2 |
| *ras*/*fos* | 0 | 4 | 0 |
| Total | 12 | 10 | 5 |

Co-overexpression of oncogene products was calculated. For each grade of tumour, the number of samples expressing a maximum of two, or three or four oncogene products is shown. Each of the combinations shown is mutually exclusive. Each of the possible combinations were sought and a zero incidence appears for those combinations that were not observed.

### *Heterogeneity in staining pattern*

A number of tissues showed heterogeneity in the staining pattern in which the silver grains were not evenly distributed over the whole of the tumour. This was most noticeable in anaplastic astrocytomas immunoreacted with anti-EGF-R and is illustrated in Figure 2. This observation suggests that there may be some clones within the tumour at any one time point in which genetic alterations are proceeding along different pathways in different regions of the tumour.

### Discussion

In contrast to previous studies which concentrated predominantly on the activation of single oncogenes in glioblastomas, this study has investigated the activation of a number of oncogenes in low, intermediate and high grade astrocytomas. The study was designed in this way to determine if a particular pattern of oncogene co-overexpression is associated with histological grade. An immunohistological technique was used to determine over-expression because the study was performed on archival material, and because this technique allows visualisation of expression in individual tumour cells and of variation in expression in different regions of the same tumour. None of these aspects is possible with DNA and RNA blotting analyses and the latter techniques also do not allow correction for the increased cellularity of tumours.

**Figure 2** Heterogeneous expression of the c-*erb*B-1 gene product in astrocytoma. Section from a single anaplastic astrocytoma was immunoreacted with an antibody against the c-*erb*B-1 gene product as described in the legend to Figure 1. Panels **a** and **b** show differences in expression of c-*erb*B-1 in two different regions and panel **c** shows variation in c-*erb*B-1 expression between tumour cells in the same region. × 1000.

We observed elevated expression of c-*erb*B-1 in all grades of astrocytomas. The percentage of tumours showing elevated expression of c-*erb*B-1 in low grade astrocytomas was 95%. Elevated expression of this oncogene in low grade astrocytomas has previously been demonstrated in only 9% of cases by Reifenberger *et al.* (1989). However their study included a number of low grade non-glial tumours in addition to low grade astrocytomas and the immunohistochemical technique used (peroxidase-anti-peroxidase) was different. Our data show that overexpression of c-*erb*B-1 in low grade astrocytomas is much higher than previously appreciated. We also observed elevated expression of c-*erb*B-1 in 86% of glioblastomas multiforme. This is comparable to data obtained by Reifenberger *et al.* (1989) who showed elevated expression in 79% of high grade astrocytomas by immunohistochemical techniques and data obtained by Malden *et al.* (1988) who showed elevated expression in 89% of glioblastoma multiforme by Northern blot analysis of total RNA. Malden *et al.* also demonstrated elevated levels of EGF-R protein in a number of those glioblastomas by Western blot analysis. Tuzi *et al.* (1991) found elevated expression of EGF-R in 29% of glial tumours but could not detect gene rearrangements in any of these cases. Interestingly cell lines derived from these tumours did not show overexpression of EGF-R. It was suggested that EGF-R amplification may confer a selective advantage *in vivo*, but not *in vitro*.

The percentage of tumours overexpressing c-*erb*B-1 was lower in anaplastic astrocytomas (48%) than in low grade tumours (95%) and glioblastoma multiforme (86%) (Table I). The reason for this observation is not clear, but may be related to the occurrence of different types of mutations in this oncogene in the different grades of tumours. It is well documented that the c-*erb*B-1 gene often undergoes extensive amplification and rearrangement in glioblastoma multiforme as a result of which the extracellular portion of the molecule is deleted (Liberman *et al.*, 1985; Malden *et al.*, 1988; Wong *et al.*, 1987). The anti-EGF-R monoclonal antibody used is directed against the extracellular portion of the molecule. One interpretation of our data would therefore be that c-*erb*B-1 overexpression occurs in all gliomas but rearrangement of the gene resulting in the truncation of the EGF-R molecule is more common in anaplastic astrocytomas than in glioblastoma multiforme.

Elevated expression of the c-*myc* oncogene has been reported in isolated glial tumours (Englehard *et al.*, 1989; Trent *et al.*, 1986), and there is a single report of elevated expression of N-*ras* in five glioblastomas (Gerosa *et al.*, 1988). Our data show that elevated expression of both c-*myc* and Ha/N-*ras* is a common feature of anaplastic astrocytomas and glioblastoma multiforme. Elevated expression of c-*fos* in astrocytomas has not been reported to our knowledge. Our data show that overexpression of this oncogene occurs in approximately 50% of all grades of astrocytoma.

A study aimed at identifying markers of tumour progression would ideally require the analysis of serial samples from the same set of patients. However ethical and practical considerations make this approach impossible in the case of astrocytic tumours. It is therefore necessary to draw conclusions from a cross-sectional study. A statistically significant difference in overexpression of c-*erb*B-1 was found between low grade tumours, anaplastic astrocytomas and glioblastoma multiforme. However, we are not able to conclude that overexpression of c-*erb*B-1 is associated with progression since 95% of low grade tumours showed overexpression of this oncogene. The low grade astrocytomas in this study were of the juvenile pilocytic type in which progression occurs very rarely (Wallner *et al.*, 1988). It is to be noted that in a study of EGF-R activity in intracranial tumours, Hawkings *et al.* (1991) also concluded that EGF-R was of little prognostic significance. Overexpression of c-*fos* was found in approximately equal numbers of low grade tumours, anaplastic astrocytomas and glioblastoma multiforme and similarly does not appear to be significantly associated with any one grade of astrocytoma or with progression. An interesting finding was that 11 of 20 low grade tumours overexpressed the combination of c-*erb*B1 and c-*fos*. This combination was not observed in anaplastic astrocytomas and was found in only one glioblastoma multiforme. This suggests that tumours found to be expressing the combination c-*erb*B-1/c-*fos* are more likely to be low grade and that they are unlikely to progress. On the other hand the proportion of samples overexpressing c-*myc* or Ha/N-*ras* increased with tumour grade. A test for trend in proportions, which is the more accurate statistical method to determine the significance of the increase observed across the three tumour grades showed this increase to be highly significant in each case, which strongly suggests that these two oncogenes are good candidates as markers of progression. Expression of the c-*myc* oncogene, has been shown to be related to the cell cycle. During the G0 phase there is a low level of c-*myc* expression. When cells are stimulated to divide, endogenous c-*myc* levels increase as cells progress from the G0 to the G1

and S phases of the cell cycle (Cole, 1986). Enhanced expression of c-*myc* in astrocytic tumours may simply be an indication that a higher proportion of cells are in a proliferative phase compared with normal brain, but detection of this at any one time point may be an important indication of the likelihood of tumour progression.

Despite the relatively small number of tumours examined, our data strongly suggest that there is an increase in the number of oncogenes overexpressed concurrent with an increase in the severity of the tumour. Glioblastoma multiforme overexpressed more oncogenes than anaplastic and low grade astroctyomas. The current hypothesis regarding the molecular basis of tumour progression implies that oncoproteins act at all levels of signal transduction and that their deregulated expression drives cell proliferation (Hunter, 1991). Recent evidence strongly suggests that sequential inactivation of oncogenes occurs hand-in-hand with loss of putative tumour suppressor genes (Marshall, 1991) and that both phenomena contribute to tumour initiation and progression. It is possible that overexpression of at least some of the four oncogenes in our study is a consequence of chromosomal aberrations and that their gene products may not work in collaboration to generate the tumour phenotype. On the other hand our data is in agreement with observations that mutational events do not have to occur in a particular sequence to promote tumour progression (Hunter, 1991) and that it is the total accumulation of mutations which is the crucial factor in determining progression to malignancy.

The authors would like to express their thanks to Mr R. Lau, Mr F. Feleppa, Ms R. Lazano and Mr A. Anile for technical assistance and Mr B. Kreunnen for photography. We also gratefully acknowledge the assistance of Penelope Jones and Caroline Finch for statistical analysis of the data and Dr Anthony W. Burgess for useful discussions during the preparation of this manuscript.

**References**

ARMITAGE, P. & BERRY, G. (1987). In *Statistical Methods in Medical Research*. 2nd ed. pp 372–374. Blackwell Scientific Publications: London.

BECKER, L.E. & YATES, A.J. (1986). Astrocytic tumours in children. In *Pathology of Neoplasia in Children and Adolescents*. Finegold, M. (ed), pp 373–396. Philadelphia: W.B. Saunders Company.

BARTRAM, C.R. & BERTHOLD, F. (1987). Amplification and expression of the N-*myc* gene in neuroblastoma. *Eur. J. Pediatr.*, **146**, 162–165.

BLIN, N., MULLER-BRECHLIN, R., CARSTENS, C., MEESE, E. & ZANG, K.D. (1987). Enhanced expression of four cellular oncogenes in a human glioblastoma cell line. *Cancer Genet. Cytogenet.*, **25**, 285–292.

BORG, A., LINELL, F., IDVALL, I., JOHANSSON, S., SIGURDSSON, H., FURNO, M. & KILLANDER, D. (1989). HER-2/neu amplification and comedo type breast carcinoma. *Lancet*, **8649**, 1268–1269.

BRODEUR, G.M. (1990). Neuroblastoma – clinical applications of molecular parameters. *Brain Pathol.*, **1**, 47–54.

COLE, M.D. (1986). The myc oncogene: its role in transformation and differentiation. *Ann. Rev. Genet.*, **20**, 361–384.

CURRAN, T., MILLER, A.D., ZOKAS, L. & VARDA, I.M. (1984). Viral and cellular fos proteins: a comparative analysis. *Cell*, **36**, 259–268.

CURRAN, T., VAN BEVEREN, C., LING, N. & VERMA, I.M. (1985). Viral and cellular fos proteins are complexed with a 39,000-dalton cellular protein. *Mol. Cell. Biol.*, **5**, 167–173.

DALLA-FAVERA, R. & CESARMAN, E. (1986). Cellular oncogenes and the pathogenesis of human cancer. In Blasi, F. (ed.), *Human Genes and Diseases*, pp 503–544. New York: John Wiley and Sons.

ENGLEHARD, H.H., BUTLER, A.B. & BAUER, K.D. (1989). Quantification of the c-myc oncoprotein in human glioblastoma cells and tumour tissue. *J. Neurosurg.*, **71**, 224–232.

EVAN, G.I. & HANCOCK, D.C. (1985). Studies on the interaction of the human c-*myc* protein with cell nuclei: p62-c-*myc* as a member of a discrete subset of nuclear proteins. *Cell*, **43**, 253–261.

FUKUI, M., YAMAMOTO, T., KAWAI, S., MITSONUBU, F. & TOYOSHIMA, K. (1987). Molecular cloning and characterization of an activated human c-raf-1 gene. *Mol. Cell. Biol.*, **7**, 1776–1781.

FUJIMOTO, M., SHERIDAN, P.J., SHARP, Z.D., WEAKER, F.J., KAGAN-HALLET, K.S., & STORY, J.L. (1989). Proto-oncogene analyses in brain tumors. *J. Neurosurg.*, **70**, 910–915.

GARSON, J.A., MCINTYRE, P.G. & KEMSHEAD, J.T. (1985). N-myc amplification in malignant astrocytoma. *Lancet*, **8457**, 718–719.

GEROSA, M.A., TALARICO, D.T., FOGNANI, C., RAIMONDI, E., COLOMBATTI, M., TRIDENTE, G., DE CARLI, L. & DELLA VALLE, G. (1988). Over-expression of N-*ras* oncogene and epidermal growth factor receptor gene in human glioblastomas. *J. Natl Cancer Inst.*, **81**, 63–67.

HARLOW, E. & LANE, D. (1988). *Antibodies. A Laboratory Manual*, pp 528–538. Cold Spring Harbour Laboratory: N.Y.

HAWKINS, R.A., KILLEN, E., WITTLE, I.R., JACK, W.J.L., CHETTY, H. & PRESCOTT, R.J. (1991). Epidermal growth factor receptors in intracranial and breast tumours: their clinical significance. *Br. J. Cancer*, **63**, 553–560.

HENN, W., BLIN, N. & ZANG, K.D. (1986). Polysomy of chromosome 7 is correlated with overexpression of the erbB oncogene in human glioblastoma cell lines. *Human Genet.*, **74**, 104–106.

HOLGATE, C.S., JACKSON, P., COWEN, P.N. & BIRD, C.C. (1983). Immunogold-silver staining: new method of immunostaining with enhanced sensitivity. *J. Histochem. Cytochem.*, **31**, 938–944.

HUNTER, T. (1991). Cooperation between oncogenes. *Cell*, **64**, 249–270.

KINZLER, K.W., ZEHNBAUER, B.A., BRODEUR, G.M., SEEGER, R.C., TRENT, J.M., MELTZER, P.S. & VOGELSTEIN, B. (1986). Amplification units containing human N-myc and c-myc genes. *Proc. Natl Acad. Sci. USA*, **83**, 1031–1035.

KINZLER, K.W., BIGNER, S.H., BIGNER, D.D., TRENT, J.M., LAW, M.L., O'BRIEN, S.J., WONG, A.J. & VOGELSTEIN, B. (1987). Identification of an amplified, highly expressed gene in a human glioma. *Science*, **236**, 70–73.

LACAL, J.C. & TRONICK, S.R. (1988). The *ras* oncogene. In Reddy, E.P., Salka, A.M. & Curran, T. (eds), *The Oncogene Handbook*, pp 254–304. Amsterdam: Elsevier Science Publishers.

LIBERMANN, T.A., NUSBAUM, H.R., RAZON, N., KRIS, R., LAX, I., SOREQ, H., WHITTLE, N., WATERFIELD, M.D., ULLRICH, A. & SCHLESSINGER, J. (1985). Amplification, enhanced expression and possible rearrangement of EGF receptor gene in primary human brain tumours of glial origin. *Nature*, **313**, 144–147.

MAGUIRE, H.C. & GREENE, M.I. (1989). The *neu* (c-*erb*B-2) oncogene. *Semin. Oncol.*, **16**, 148–155.

MALDEN, L.T., NOVAK, U., KAYE, A.H. & BURGESS, A.W. (1988). Selective amplification of the cytoplasmic domain of the epidermal growth factor receptor gene in glioblastoma multiforme. *Cancer Res.*, **48**, 2711–2714.

MARSHALL, C.J. (1991). Tumour suppressor genes. *Cell*, **64**, 313–326.

NAKAGARAWA, A., IKEDA, K., TSUDA, T., HIGASHI, K. & OKABE, T. (1987). Amplification of N-myc oncogene in stage II and IVS neuroblastomas may be a prognostic indicator. *J. Pediatr. Surg.*, **22**, 415–418.

REIFENBERGER, G., PRIOR, R., DECKER, M. & WESCHLER, W. (1989). Epidermal growth factor receptor expression and growth fraction in human tumours of the nervous system. *Virchows Archiv. A Pathol. Anat.*, **414**, 147–155.

RINGERTZ, N. (1950). Grading of gliomas. *Acta Pathol. Microbiol. Immunol. Scand.*, **27**, 51–54.

SLAMON, D.J., CLARK, G.M., WONG, S.G., LEVIN, W.J., ULLRICH, A. & MCGUIRE, W.L. (1987). Human breast cancer: correlation of relapse and survival with amplification of the HER-2/*neu* oncogene. *Science*, **235**, 177–182.

TRENT, J., MELTZER, P., ROSENBLUM, M., HARSH, G., KINZLER, K., MARSHAL, R., FEINBERG, A. & VOGELSTEIN, B. (1986). Evidence for rearrangement, amplification, and expression of c-myc in a human glioblastoma. *Proc. Natl Acad. Sci. USA*, **83**, 470–473.

TSUDA, T., OBARA, M., HIRANO, H., GOTOH, S., KOBOMURA, S., HIGASHI, K., KUROIWA, A., NAKAGARAWA, A., NAGAHARA, N. & SHIMIZU, K. (1987). Analysis of N-myc amplification in relation to disease stage and histologic types in human neuroblastomas. *Cancer*, **60**, 820–826.

TUZI, N.L., UENTER, D.J., KUMAR, S., STADDON, S.L., LEMOINE, N.R. & GULLICK, W.J. (1991). Expression of growth factor receptors in human brain tumours. *Br. J. Cancer*, **63**, 227–233.

VAN DE VIJVER, M.J., PETERSE, J.L., MOOI, W.J., WISMAN, P., LOMANS, J., DALESIO, O. & NUSSE, R. (1988). *Neu*-protein over expression in breast cancer. Association with comedo-type ductal carcinoma *in situ* and limited prognostic value in stage II breast cancer. *New England J. Med.*, **319**, 1239–1245.

WALLNER, K.E., GONZALES, M.F., EDWARDS, M.S.B., WARA, W.M. & SHELINE, G.E. (1988). Treatment results of juvenile pilocytic astrocytoma. *J. Neurosurg.*, **69**, 171–176.

WONG, A.J., BIGNER, S.H., BIGNER, D.D., KINZLER, K.W., HAMILTON, S.R. & VOGELSTEIN, B. (1987). Increased expression of the epidermal growth factor receptor gene in malignant gliomas is invariably associated with gene amplification. *Proc. Natl Acad. Sci. USA*, **84**, 6899–6903.

WU, J.K. & CHIKARAISHI, D.M. (1990). Differential expression of ros oncogene in primary human astrocytomas and astrocytoma cell lines. *Cancer Res.*, **50**, 3032–3035.

YEATON, R.W., LIPARI, M.T. & FOX, C.F. (1983). Calcium-mediated degradation of epidermal growth factor receptor in dislodged A431 cells and membrane preparations. *J. Biol. Chem.*, **258**, 9254–9261.

ZULCH, K.J. (1986). In *Brain Tumours. Their Biology and Pathology*, pp 1–26. Berlin: Springer–Verlag.